# Spontaneous bilateral superficial femoral artery pseudoaneurysms and a unilateral posterior tibial artery aneurysm in an immunocompromised patient

**DOI:** 10.1002/ccr3.8686

**Published:** 2024-03-20

**Authors:** Jonathan Crisp, Manal Ahmad, Stephen Crockett, Abdulla Mohamed, Mohamad Hamady, Ondina Bernstein, Joseph Shalhoub

**Affiliations:** ^1^ Department of Vascular Surgery Imperial College Healthcare NHS Trust London UK; ^2^ Department of Surgery and Cancer Imperial College London London UK; ^3^ Department of Radiology Imperial College Healthcare NHS Trust London UK

**Keywords:** aneurysm, endovascular, immunodeficiency, pseudoaneurysm, vascular

## Abstract

**Key Clinical Message:**

The presence of multiple pseudoaneurysms in a patient should prompt investigations for the underlying etiologies including autoimmune and immunosuppressive disease processes. Treatment options include open repair and endovascular stenting.

**Abstract:**

Pseudoaneurysms (also known as false aneurysms) are atypical dilatations or outpouchings from a vessel which are not always contained by the three layers of a normal vessel wall, namely the intima, media, and adventitia. These are distinct from a true aneurysm which has a wall comprising all three layers. The underlying etiology for both true aneurysms and pseudoaneurysm can vary. We present the rare case of bilateral superficial femoral artery pseudoaneurysms, of unknown etiology and a concurrent posterior tibial artery saccular aneurysm in a patient with Human Immunodeficiency Virus (HIV) infection and multiple comorbidities. This was managed using a combination of endovascular covered stent grafts and open surgical repair technique. The patient is doing well on follow‐up a year later with no post‐operative infections. A literature review of the existing reports of superficial femoral artery pseudoaneurysms and posterior tibial artery aneurysms and their management is also reported.

## Graphical abstract

Pre‐operative CT imaging of pseudoaneurysms (A+E). Digital subtraction angiography of pseudoaneurysm prior to stenting (B+F) and post stenting (C+G). Post‐operative CT imaging (D+H).

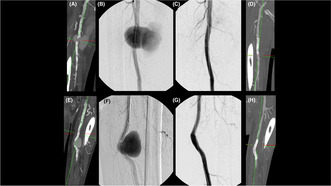

## 1 INTRODUCTION

Pseudoaneurysms (also known as false aneurysms) are atypical dilatations or outpouchings from a vessel which are not always contained by the three layers of a normal vessel wall, namely intima, media, and adventitia. These are distinct from a true aneurysm which has a wall comprising all three layers.

Pseudoaneurysms are most commonly a result of trauma but can also be iatrogenic during catheterization of vessels, vessel instrumentation, at anastomotic sites or due to failure of percutaneous closure devices.^1^ Spontaneous superficial femoral artery (SFA) pseudoaneurysms are rare, but have been reported to be associated with infection, vasculitides, and connective tissue disorders.^2^

We present this unique case where the SFA pseudoaneurysms, were bilateral, spontaneous, without any obvious infective cause with an associated synchronous saccular aneurysm of the left posterior tibial artery (PTA), which together has not been reported as a case in the literature to date.

## 2 CASE PRESENTATION

A male immunocompromised patient in his early 40's presented with a swelling in his right anterior thigh. This had gradually become painful over the preceding 2 weeks becoming particularly symptomatic whilst attending for hemodialysis on the day of presentation. He had no preceding symptoms of peripheral arterial disease.

The patient's extensive past medical history included type 1 diabetes, end stage renal disease secondary to diabetic nephropathy requiring hemodialysis, diabetic retinopathy, autoimmune hypothyroidism, hypertension, human immunodeficiency virus (HIV) with a controlled viral load, ischemic stroke, deep venous thrombosis, pulmonary embolism, mitral regurgitation, endocarditis, dilated cardiomyopathy, and COVID‐19 pneumonitis 3 years earlier. He was not known to have peripheral arterial disease.

The patient resided at home with his family and was supported by his mother and a social care package three times a day and mobilized in a wheelchair. He had never smoked but had an extensive history of alcohol intake in the past. There was no history of intravenous drug use or use of illicit substances. There was no associated history of trauma or triggering events prior to the development of his symptoms. The contralateral leg remained asymptomatic. Two days prior to presentation the patient had been started on a course of amoxicillin to treat a presumed community acquired pneumonia (CAP). In addition, the patient had recently been admitted to hospital 4 months prior with a lower respiratory tract infection and had required admission to the intensive care unit. All cultures including blood and sputum were negative during that admission and investigations looking for *Pneumocystis jirovecii* were also negative. Routine swabs had grown non‐specific yeasts and *Klebsiella* which were treated with Fluconazole and Ceftriaxone, respectively. The patient was anticoagulated with warfarin for a recent pulmonary embolism. He also took clopidogrel and several other medications including antiretrovirals.

On arrival, he was found to be hypoxic with bi‐basal crackles upon chest auscultation and bilateral ankle swelling, for which he was receiving oxygen therapy (15 L) but was not on long term oxygen therapy prior to his presentation. Examination of the lower limbs did not identify any abdominal masses or pulsatile masses indicative of an abdominal aortic aneurysm. He had palpable femoral pulses but absent popliteal and pedal pulses. The feet were warm and well perfused with a capillary refill time of <3 s. The right lower limb was swollen with a tender, pulsatile swelling noted medially in the right mid‐thigh in addition to a non‐tender pulsatile swelling in the left medial gaiter region. He was neurologically intact and did not have any paraesthesia or motor changes in the lower limb.

## 3 INVESTIGATIONS

**FIGURE 1:**
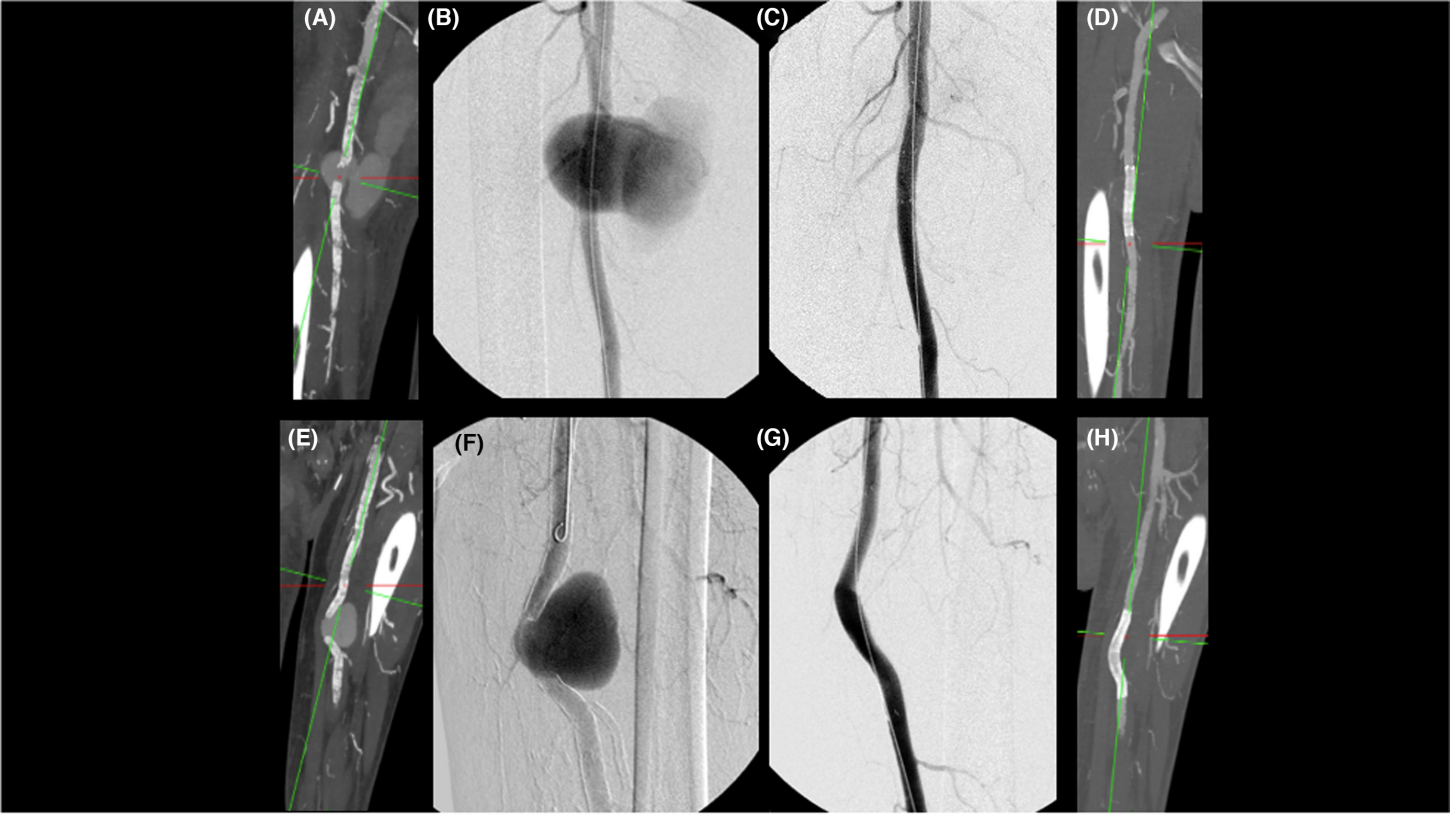
Preoperative computed tomography (CT) angiographic (A) and intraoperative digital subtraction angiographic (B) images demonstrating the ruptured right superficial femoral artery pseudoaneurysm. Intraoperative digital subtraction angiographic (C) and postoperative CT angiographic (D) images demonstrating Viabahn stent graft exclusion of the ruptured right superficial femoral artery pseudoaneurysm. Preoperative CT angiographic (E) and intraoperative digital subtraction angiographic (F) images demonstrating the left superficial femoral artery pseudoaneurysm. Intraoperative digital subtraction angiographic (G) and postoperative CT angiographic (H) images demonstrating Viabahn stent graft exclusion of the left superficial femoral artery pseudoaneurysm.

This patient came in out of hours and overnight, so imaging via a Doppler ultrasound was unavailable. A computed tomography (CT) angiogram was arranged urgently of the lower limbs. This demonstrated heavily calcified arteries throughout the lower extremities, a 6.5 cm SFA pseudoaneurysm on the right side with surrounding hematoma within the adductor canal (Figure 1A). On the left, a 4.5 cm SFA pseudoaneurysm was observed (Figure 1E). The defect size was 15 mm on the right and 19 mm on the left.

His blood tests demonstrated a hemoglobin of 92 g/L and an international normalized ratio (INR) of 1.7 (after Vitamin K). His white cell count was 7.9 × 10^9^/L and a C‐reactive protein of 53.4 mg/L which lowered the suspicion for an acutely infective cause of his vascular pathology. His last HIV‐1 viral load was 30 copies/mL. He had multiple blood cultures which were all negative, however false negative antibiotic induced results cannot be ruled out in the context of the patient having recently been commenced on antibiotics for his chest infection.

During his admission, the patient was also investigated for other potential etiological factors including a vasculitis screen, Hepatitis B and C screen all of which were negative. The patient's viral load was <20 copies/mL throughout this admission and post‐operatively. His absolute CD4^+^ was 26% prior to his presentation.

The patient's echocardiogram 6 months prior to his admission had demonstrated a severely dilated left ventricle and impaired right ventricular function, moderate tricuspid regurgitation and a pulmonary artery systolic pressure of 66 mmHg. Given that no active acute bleeding was noted on CT, he was transferred to the intensive care for hemofiltration to manage his fluid overload, hyperkalemia (potassium 5.8 mmol/L) and to optimize the patient for intervention. This fluid overload and hyperkalemia was present despite the patient having hemodialysis on the previous day, and not having received any intravenous fluid or increased his oral fluid intake. This was also discussed with the anesthetic team and with hematology who recommended Vitamin K to allow the INR to normalize and the clotting to be optimal prior to the intervention.

## 4 MANAGEMENT

A multidisciplinary team discussion opted for endovascular treatment under spinal and local anesthetic, with the patient not fully flat to avoid desaturation secondary to his ongoing pulmonary edema. The right common femoral artery was anterogradely punctured and an 8‐French sheath sited. 9 mm × 50 mm (diameter × length) followed by 10 mm × 50 mm Gore® Viabahn® endoprosthesis stent‐grafts (Flagstaff, Arizona, USA) were placed across the right SFA pseudoaneurysm, with completion angiography demonstrating successful right SFA pseudoaneurysm exclusion and a patent right SFA (Figure 1B,C).

**FIGURE 2:**
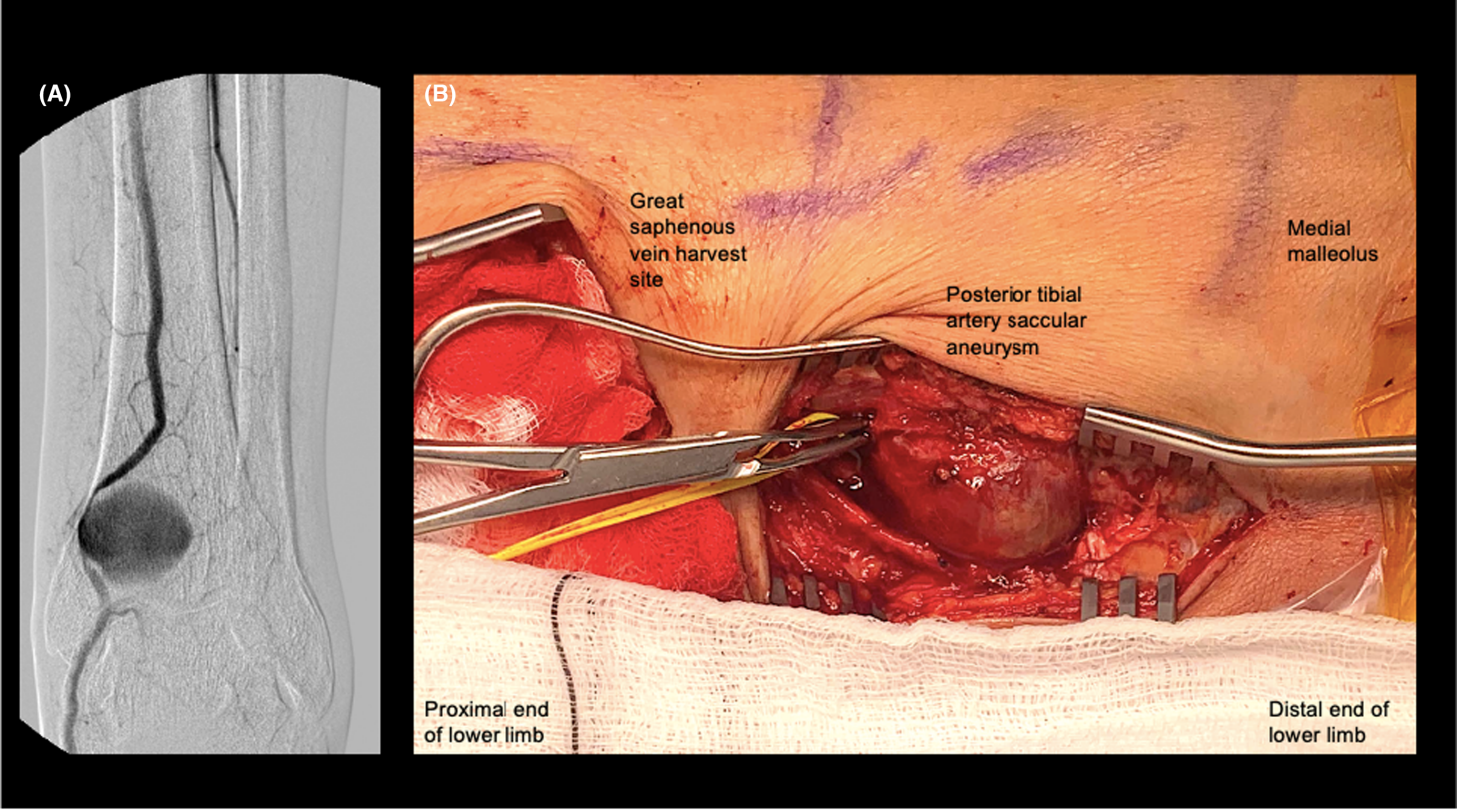
(A) Digital subtraction angiography demonstrating the left posterior tibial artery saccular aneurysm, with the posterior tibial artery being the sole inflow vessel to the foot. Intraoperative picture of the posterior tibial artery pseudoaneurysm. (B) Annotated intraoperative photograph of the exposure of the left posterior tibial artery saccular aneurysm at the time of interposition vein graft repair using reversed ipsilateral great saphenous vein, harvested from the more proximal incision.

On the left, a 10‐French sheath was required for delivery of a 11 mm diameter Viabahn stent graft and it was felt that the degree of arterial calcification would preclude safe percutaneous closure device use, a surgical cut down to the left common femoral artery was performed. The left common femoral artery was punctured under direct vision and a 10‐French sheath was sited in an antegrade fashion. An 11 mm × 100 mm Gore® Viabahn® endoprosthesis stent‐graft (Flagstaff, Arizona, USA) was placed across the left SFA pseudoaneurysm, with completion angiography demonstrating successful left SFA pseudoaneurysm exclusion and a patent left SFA (Figure 1F,G). An angiogram of the left lower limb also identified a saccular aneurysmal dilatation of the distal left PTA just proximal to the medial malleolus, with the PTA providing the sole arterial inflow to the foot (Figure 2A). Endovascular repair of the distal left PTA aneurysm, in the context of the single vessel runoff to the foot, was deemed high risk for long term patency of a distal stent and the team opted for an open repair of this once the patient had recovered from this procedure and had further medical optimization and recovery.

Postoperative CT angiography confirmed exclusion of the SFA pseudoaneurysms with patient SFA stent grafts bilaterally (Figure 1D,H). This was also confirmed on postoperative lower limb arterial duplex ultrasound which demonstrated triphasic flow throughout and thrombosed SFA pseudoaneurysms with no active flow. He was put on aspirin and treatment dose enoxaparin (for previous deep vein thrombosis). This regimen was chosen rather than warfarin and clopidogrel as pre‐op, to keep as many anesthetic options as possible prior to the next procedure. He completed a course of co‐trimoxazole to treat his community acquired pneumonia.

Four weeks later, the patient proceeded to have an open interposition repair of a saccular left posterior tibial saccular aneurysm (Figure 2B) using reversed ipsilateral distal great saphenous vein. Samples of the left PTA saccular aneurysm wall were sent for both microbiology and histopathology examination. The microbiology samples revealed no growth on extended incubation. The histopathology demonstrated a distorted vessel wall with thickened intima and thinned media and also identified and adventitia as expected with a true aneurysm. There was also coarse nodular calcification with attached luminal thrombus. No inflammation, evidence of vasculitis or features of malignancy were seen.

## 5 OUTCOME AND FOLLOW‐UP

**FIGURE 3:**
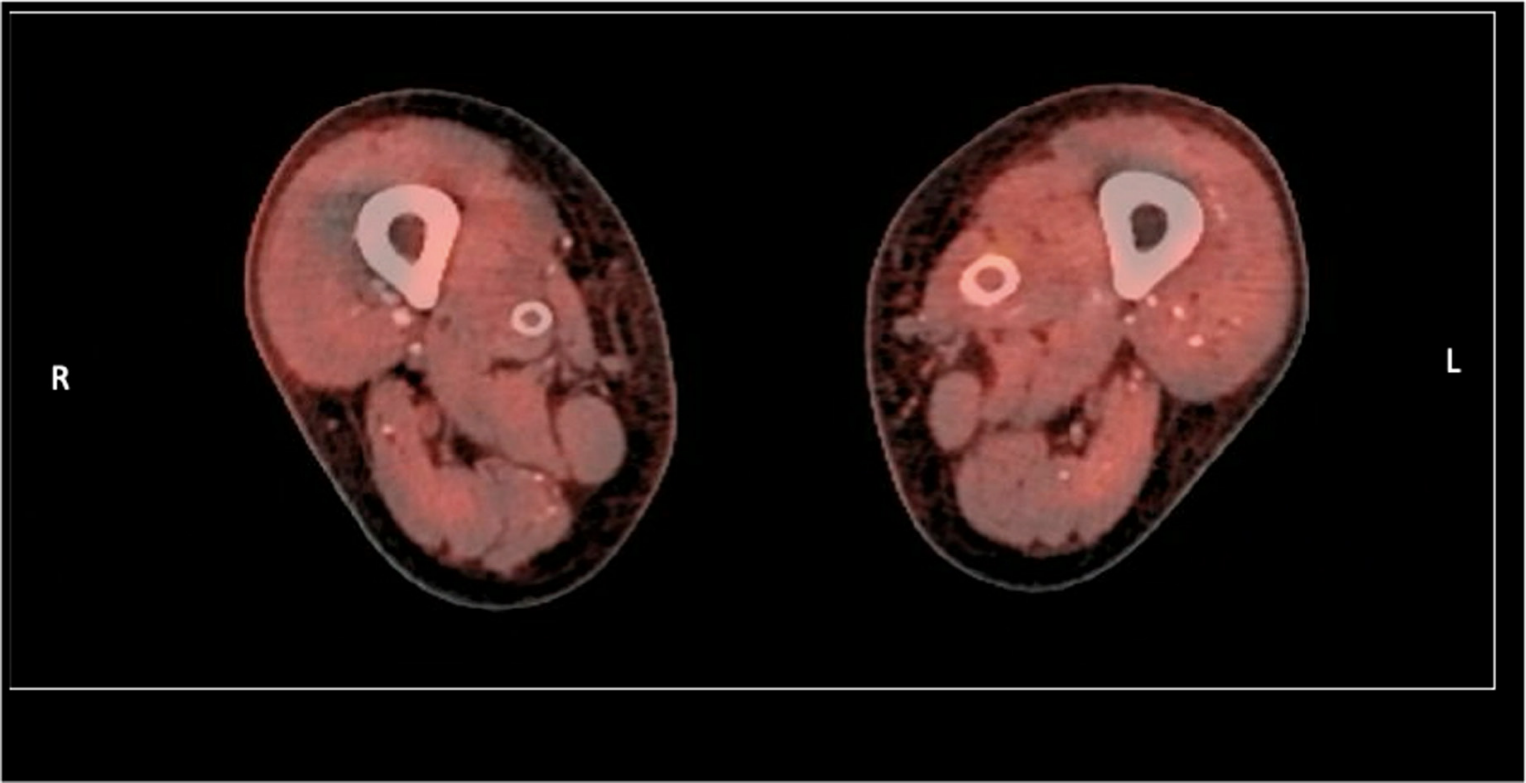
Post‐stenting and posterior tibial artery aneurysm repair Positron Emission Tomography (PET) scan 4 months later demonstrating the bilateral Viabahn stents. The PET scan confirmed that there was no increased activity related to the major vessels in the body, in the lower limbs, near the stent grafts or at the site of the pseudoaneurysms. The PET scan also helped to rule out vasculitis as an underlying cause.

Postoperatively, he had strong multiphasic signals on the handheld Doppler assessment. A follow‐up arterial duplex several weeks later demonstrated patent bilateral SFA stents with triphasic waveforms. The patient went home several days later and was seen in the clinic after 6 weeks where he was well with healed wounds and back at his usual residence. He was discharged on his usual medications which included clopidogrel and atorvastatin. A follow‐up positron emission tomography (PET) scan 4 months later was also done. The post‐stenting and posterior tibial artery aneurysm open repair confirmed that there was no increased activity related to the major vessels in the body, in the lower limbs, near the stent grafts or at the site of the pseudoaneurysms. The PET scan also further helped to exclude vasculitis as an underlying cause (Figure 3). He has been reviewed clinically on a 3‐monthly basis and is doing well clinically with no signs of infection 14 months post intervention.

## 6 DISCUSSION

A pseudoaneurysm is defined as a breach in the arterial wall of a vessel, resulting in the accumulation of blood between the tunica media and tunica adventitia of the artery and can also be contained by surrounding tissues. Due to the direct connection between the vessel lumen and the aneurysm sac, pseudoaneurysms can continue to increase in size unless they are intervened upon or spontaneously thrombose.

The etiology of pseudoaneurysm formation is variable but most etiologies involve direct damage to the arterial vessel wall. The most common causes are endovascular procedures that involve direct puncture of the arterial wall, with pseudoaneurysm thought to complicate up to 8% of interventional procedures requiring arterial puncture for access. Other common causes include trauma and intravenous drug use.^3^

Spontaneous SFA pseudoaneurysm ruptures, such as in this case, are rare,^4^ particularly when presenting bilaterally, but even more so with the presence of a third distal aneurysm. A literature review was conducted across Pubmed & Medline databases using the search terms “spontaneous”, “superficial”, “femoral artery”, “posterior tibial artery”, “pseudoaneurysm”, and “aneurysm”. We identified 11 cases of spontaneous SFA pseudoaneurysms unrelated to direct trauma or previous surgery to the affected SFA. Of these case reports, two presented with bilateral SFA pseudoaneurysms and one case presented with an SFA pseudoaneurysm of one limb & a common femoral artery (CFA) pseudoaneurysm of the contralateral leg. There were 26 cases of spontaneous PTA aneurysms identified (See Tables 1 and 2). To our knowledge, no cases of bilateral SFA pseudoaneurysms with a concurrent PTA aneurysm exists in the literature.

**TABLE 1:** Superficial femoral artery (SFA) pseudoaneurysm literature review summary table. A literature review was conducted across PubMed & Medline database using the search terms “spontaneous”, “superficial”, “femoral artery”, and “pseudoaneurysm”. Exclusion criteria included publications prior to 1990 and those not available in English.

Author	Age	Gender	Laterality	Proposed etiology	Management
Ugurlucan (2014)^5^	45	Male	Left	Behcet's disease	Interposition bypass graft
Fukunaga (2013)^6^	77	Male	Right	Hypertension, end stage renal disease	Patch repair of rupture
Siani (2008)^4^	86	Female	Left	Diffuse atherosclerosis	Endovascular–Viabahn® stent
Gunawardena (2021)^7^	51	Male	Bilateral	Diabetes, end stage renal disease	Right—bypass graft Left—conservative
Alsmady (2013)^8^	29	Male	Right	Unknown	Interposition bypass graft
Darigny (2021)^2^	17	Male	Left	Unknown	Interposition bypass graft
Samara (2013)^9^	62	Female	Left	Unknown	Endovascular–Viabahn® stent
Goh (2004)^10^	15	Male	Bilateral	Unknown	Open hematoma evacuation & ligation of avulsed artery
Kouvelos (2011)^11^	71	Female	Right	Diffuse atherosclerosis	Endovascular–stenting
Yoshida (1998)^12^	25	Male	Left	Behcet's disease	Spontaneously thrombosed
Torres‐Blanco (2016)^13^	‐	Male	Bilateral (left SFA + right CFA)	Intravesical instillation of BCG	Left—surgical resection & ligation Right—common to deep femoral artery bypass

**TABLE 2:** Posterior tibial artery (PTA) aneurysm literature review summary table. A literature review was conducted across PubMed & Medline databases using the search terms ‘spontaneous’, ‘posterior tibial artery’, and ‘aneurysm’. Exclusion criteria included publications prior to 1990 and those not available in English.

Author	Age	Gender	Laterality	Proposed etiology	Management
Sagar (2014)^14^	64	Male	Right	Unknown	Surgical excision + interposition vein graft
Beiko (2022)^15^	70	Male	Right	Acquired thrombophilia secondary to SARS‐CoV‐2	Surgical excision + end‐to‐end anastomosis
Tshomba (2006)^16^	54	Male	Not reported	Atherosclerosis	Surgical excision + end‐to‐end anastomosis
Kfoury (2017)^17^	6	Male	Right	*SMAD3* mutation	Surgical excision + end‐to‐end anastomosis
Patel (2008)^18^	47	Male	Right	Septic aneurysm secondary to endocarditis	Surgical excision + interposition vein graft
Barbano (2009)^19^	28	Male	Right	Unknown	Coil embolisation
Matsura (2021)^20^	77	Female	Right	Neurofibromatosis type 1 (NF‐1)	Coil embolisation
Tonogai (2018)^21^	55	Male	Right	Septic aneurysm secondary to endocarditis	Surgical excision + interposition vein graft
Robaldo (2012)^22^	52	Male	Right	Atherosclerosis	Surgical excision + interposition vein graft
Hattam (2019)^23^	75	Female	Bilateral	Non‐specified collagen disorder	Surgical excision + interposition vein graft
Danes (2006)^24^	60	Male	Not reported	Syphilitic infection	Surgical excision + interposition vein graft
Murakami (2011)^25^	47	Female	Left	Unknown	Surgical excision + interposition vein graft
Ferrero (2010)^26^	59	Male	Not reported	Septic aneurysm secondary to endocarditis	Surgical ligation
Sakai (2002)^27^	54	Male	Left	Unknown	Surgical excision
Plotnick (2021)^28^	38	Female	Left	Ehlers–Danlos type IV (EDS‐IV)	Endovascular flow diversion stenting
Kanaoka (2004)^29^	69	Female	Right	Atherosclerosis	Surgical excision + interposition vein graft
Menanteau (1995)^30^	78	Male	Left	Septic aneurysm secondary to endocarditis	Not reported
Hayashida (2021)^31^	30	Female	Not reported	Unknown	Surgical excision + interposition vein graft
Hagspeil (2011)^32^	24	Female	Bilateral	Ehlers–Danlos type IV (EDS‐IV)	Coil embolisation
Hasaniya (1993)^33^	32	Female	Left	Polyarteritis nodosa	Coil embolisation
Toncev (2013)^34^	61	Male	Right	Unknown	Surgical excision
McKee (1999)^35^	15	Male	Bilateral	Septic aneurysm secondary to endocarditis	Surgical excision + interposition vein graft
Chaillou (1992)^36^	62	Male	Right	Behcet's disease	Surgical excision
Singh (2018)^37^	25	Female	Right	Vasculitis of unknown origin	Surgical excision + interposition vein graft
Al Agha (2016)^38^	53	Female	Left	Unknown	Surgical excision + end‐to‐end anastomosis
Jiang (2018)^39^	65	Male	Left	Septic aneurysm secondary to endocarditis	Not stated

A number of cases in individuals of similar age to our patient were linked to vasculitides such as Behcet's disease^5^, ^12^, ^36^ whereby vascular inflammation and endothelial dysfunction lead to a deterioration in arterial structure, increasing the risk of pseudoaneurysm formation, however the vasculitis screen came back negative for our patient. In patients with diabetes, as in our case, pseudoaneurysm formation can occur through immunocompromise and overall tissue glycation. Pro‐atherosclerotic risk factors lead to direct maladaptive changes in the arterial wall microstructure that facilitates pseudoaneurysm formation.^7^, ^13^

The patient's HIV status alongside their extensive cardiovascular risk factors are likely contributing factors to pseudoaneurysm formation although the underlying definitive etiology was not confirmed. There are numerous studies that postulate HIV infection directly leads to arterial wall weakening,^40^, ^41^ and several case reports hypothesizing that a HIV vasculopathy may have directly resulted in pseudoaneurysm formation in other anatomical locations.^42^, ^43^, ^44^, ^45^ Arterial endothelial cells are continually exposed to mechanical stimuli such as hemodynamic forces and fluid shear stress. Sustained stimuli lead to remodeling of cellular cytoskeletal structures and activate intracellular signaling pathways which are adaptive and maintain vascular hemostasis. When these processes are perturbed by excessive hemodynamic and inflammatory stimuli, triggered by factors such as HIV infection, hypertension and diabetes, they become pathological. This reduces nitric oxide bioavailability, increases reactive oxygen species production and changes adhesion molecule expression. In turn, this causes endothelial cell dysfunction and intimal fragility, predisposing to aneurysm formation.^46^

Mycotic aneurysm formation secondary to endocarditis is another frequent explanation for aneurysm formation in several cases in the literature.^18^, ^21^, ^26^, ^30^, ^39^ The patient had been started on oral amoxicillin 2 days prior to transfer to our hospital. This means there is the possibility that his negative blood cultures were antibiotic false negative cultures, although the multiple samples that were sent would make this unlikely.

Pseudoaneurysm management techniques vary from conservative approaches, to open, endovascular or hybrid open‐endovascular repair techniques. The most appropriate choice of treatment is case‐specific and relies on numerous factors such as patient age, comorbidities, pseudoaneurysm anatomy and etiology, and endovascular treatment availability.^3^ Possible management options for pseudoaneurysms include ultrasound‐guided thrombin injection, endovascular repair using stent grafts, or open surgical repair.^3^ An endovascular approach was chosen for this patient as his SFA pseudoaneurysm anatomy allowed for endovascular stent graft insertion which was less invasive, considering his comorbidities and clinical status at time of presentation. An open reconstruction was conducted for the patient's PTA saccular aneurysm due to concerns that endovascular exclusion could compromise the sole arterial flow into the foot. This was performed in a non‐emergency setting, after the patient had recovered from their acute cardio‐respiratory decompensation and had been medically optimized to reduce his overall anesthetic risk.

## AUTHOR CONTRIBUTIONS

**Jonathan Crisp:** Data curation; writing – original draft. **Manal Ahmad:** Resources; writing – original draft; writing – review and editing. **Stephen Crockett:** Writing – original draft; writing – review and editing. **Abdulla Mohamed:** Writing – review and editing. **Mohamad Hamady:** Investigation; visualization. **Ondina Bernstein:** Investigation; visualization. **Joseph Shalhoub:** Supervision; writing – review and editing.

## FUNDING INFORMATION

Funding for this review was provided by the Imperial College London's Section of Vascular Surgery NIHR Imperial Biomedical Research Centre (BRC) fund for infrastructural support.

## CONFLICT OF INTEREST STATEMENT

The review authors have no competing interests to declare.

## CONSENT

Written informed consent was obtained from the patient to publish this report in accordance with the journal's patient consent policy.

## Data Availability

All data generated or analyzed during this study are included in this published article [and its supplementary information files].
